# Micro Optical Coherence Tomography for Coronary Imaging

**DOI:** 10.3389/fcvm.2021.613400

**Published:** 2021-03-26

**Authors:** Kensuke Nishimiya, Guillermo Tearney

**Affiliations:** ^1^Wellman Center for Photomedicine, Massachusetts General Hospital, Boston, MA, United States; ^2^Department of Cardiovascular Medicine, Tohoku University Graduate School of Medicine, Sendai, Japan; ^3^Department of Pathology, Massachusetts General Hospital, Boston, MA, United States; ^4^Harvard-Massachusetts Institute of Technology (MIT) Division of Health Sciences and Technology Division, Cambridge, MA, United States

**Keywords:** optical coherence tomography, micro-OCT, endothelial cells, inflammatory cells, macrophage—cell, cholesterol crystals, necrotic core, plaque erosion

## Abstract

Intravascular optical coherence tomography (IVOCT) that produces images with 10 μm resolution has emerged as a significant technology for evaluating coronary architectural morphology. Yet, many features that are relevant to coronary plaque pathogenesis can only be seen at the cellular level. This issue has motivated the development of a next-generation form of OCT imaging that offers higher resolution. One such technology that we review here is termed micro-OCT (μOCT) that enables the assessment of the cellular and subcellular morphology of human coronary atherosclerotic plaques. This chapter reviews recent advances and ongoing works regarding μOCT in the field of cardiology. This new technology has the potential to provide researchers and clinicians with a tool to better understand the natural history of coronary atherosclerosis, increase plaque progression prediction capabilities, and better assess the vessel healing process after revascularization therapy.

## μOCT: Beyond Standard OCT

In the early 1990's, intravascular optical coherence tomography (IVOCT) (1) commenced with the understanding that OCT (2) could be clinically applied beyond ophthalmology. Conventional IVOCT employs broadband near-infrared light centered at a wavelength of 1,300 nm (3), providing it with a spatial resolution of about 10 μm that is an order of magnitude higher than that of intravascular ultrasound (IVUS) (4). The roughly 10-μm-resolution of IVOCT provides detailed information on treated and untreated coronary plaque morphology by resolving varying arterial microscopic architectural structures (5–10). Similarly to the circumferential view of IVUS, depth information provided by IVOCT makes it possible to display coronary artery lumen cross-sections (9, 10), luminal narrowing (11), and intimal thickening (12). Its higher resolution enables other features to be clearly identified, including fibrous cap thickness (4–6, 13), lipid (4, 13), cholesterol crystals (4), thrombus (4), dissections (4), macrophage accumulations (4, 7), calcium (4–6), and intimal neo-vasculature (4). Hence, IVOCT has improved diagnostic accuracy for human coronary plaques (5, 7), and its feasibility for guiding coronary intervention has been consistently demonstrated (14–19). Over the past two decades, interventional cardiologists and engineers have worked together to make tremendous progress to develop and validate IVOCT as a useful instrument for visualizing the detailed morphology of coronary plaque and stents.

Despite the potential importance of physiological assessment of myocardial ischemia due to significant organic coronary stenosis (20), recent studies have highlighted that the initial interventional strategy with percutaneous coronary intervention or coronary artery bypass does not necessarily result in better clinical outcomes in stable (chronic) coronary artery disease (CAD) patients when compared to optimal medical therapy (21). These results have raised questions regarding what coronary morphological features bestow high risk of a future clinical event, regardless of the severity of the luminal narrowing. To address these questions, OCT with even higher resolution could illuminate the roles of coronary microstructures heretofore unseen, such as individual coronary endothelial cells (22, 23), inflammatory cells, cholesterol crystals (24), vascular smooth muscle cells (25), fibroblasts, (micro-)calcifications (26), and components of thrombi such as platelets and fibrin, all thought to play roles in natural history of coronary atherosclerosis and the clinical manifestations of high risk lesions (27).

In 2011, a new mode of OCT termed micro-OCT (μOCT) was demonstrated with a resolution of 1–2 μm (28). The initial μOCT technology was implemented using a bench-top microscope system and has shown broad utility for a variety of *in vitro* and *ex vivo* studies and applications (28–32). Recently, to implement μOCT clinically, a single fiber optic μOCT probe and intracoronary catheter have been created (33, 34)—the technology is now poised to be used in coronaries *in vivo* (35). In this article, we review the developments in μOCT technology and describe its potential clinical implications for intracoronary imaging.

## μOCT for Coronary Endothelial Cell Visualization

Endothelial cells act as gatekeepers for the passage of low-density lipoprotein (LDL) and leukocytes into the intima, and thus endothelial disruption/dysfunction is considered to be an important catalyst of coronary atherogenesis (36, 37). Previous ultrastructural studies have demonstrated that endothelial cells cover the intima in a “cobblestone” pattern, also known as “endothelial pavementing” on *en-face* SEM (22). It has been recognized that coronary plaque erosion characterized by lesions with loss of endothelial cells beneath thrombus is the second most prevalent histopathological cause of acute coronary syndrome (ACS) (27, 38, 39).

**Figure 1:**
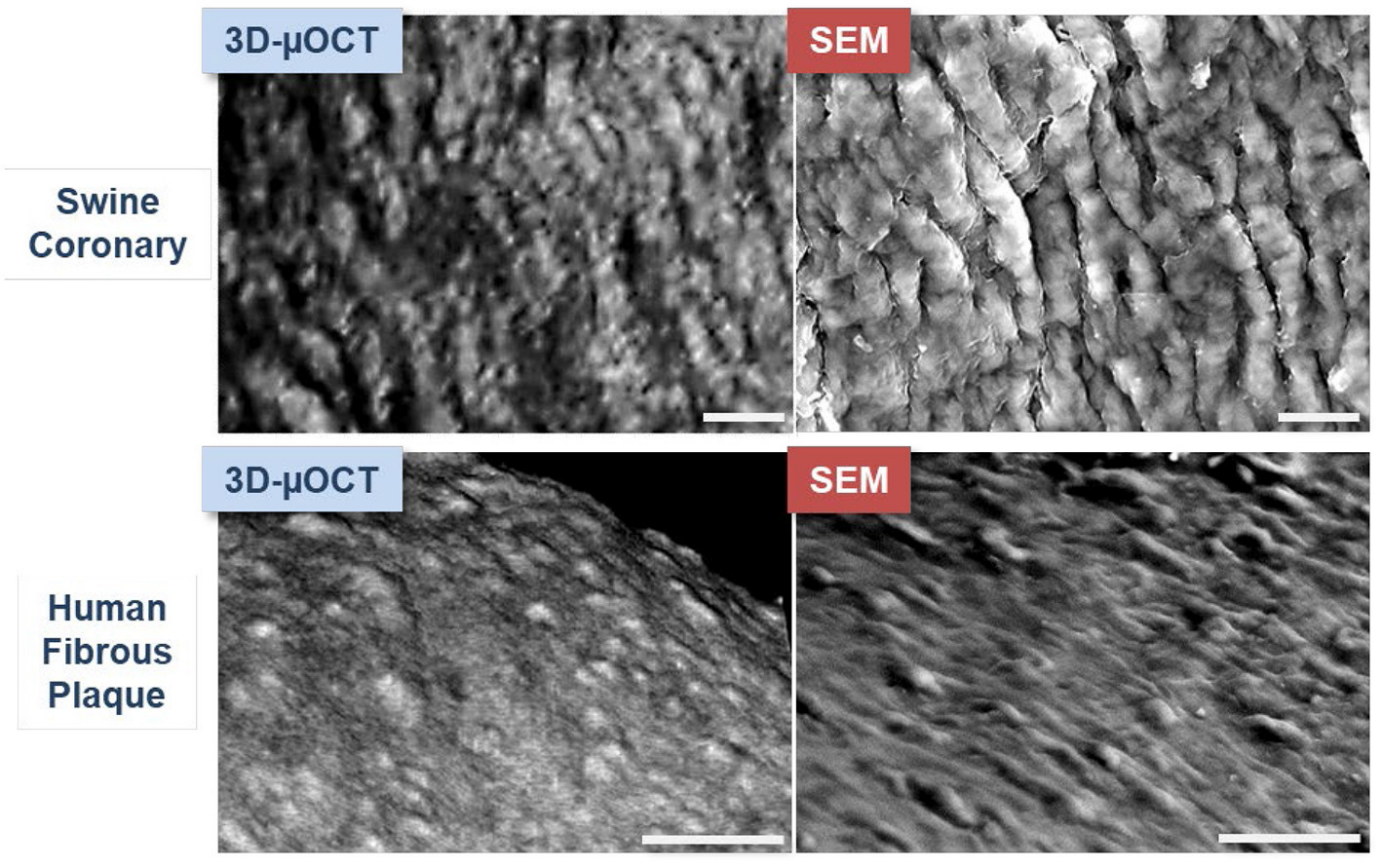
μOCT images of endothelial pavementing and corresponding SEM in swine and human coronary arteries *ex vivo*. Upper panels: Swine coronary endothelial pavementing visualized by three-dimensionally (3D) volume-rendered μOCT is similar to that seen by scanning electron microscopy (SEM). Lower panels: Findings were consistent in human coronary fibrous plaque. Scale bars, 25 μm. Figure and capture reprinted with permission from Nishimiya et al. (40).

μOCT has been shown to visualize swine and human coronary endothelial cells *ex vivo* (40). The capability of μOCT to visualize endothelial cells was validated with the current gold standard scanning electron microscopy (SEM) (22). The histological validation study included a visual comparison of swine coronary endothelial pavementing seen by μOCT, volume-rendered in three-dimension (3D-μOCT), with that seen by *en-face* SEM (Figure 1) (40). 3D-μOCT images clearly showed the uneven endothelial surface corresponding to coronary endothelial pavementing seen on corresponding SEM. After endothelial stripping (41), the surface roughness disappeared from the 3D-μOCT image, indicating the absence of endothelial cells. Quantitative analysis was performed by calculating surface roughness on a μOCT data-set and the corresponding SEM (40), demonstrating a high degree of correlation between μOCT and the SEM gold standard (*R*^2^ = 0.99, *P* < 0.01).

**Figure 2:**
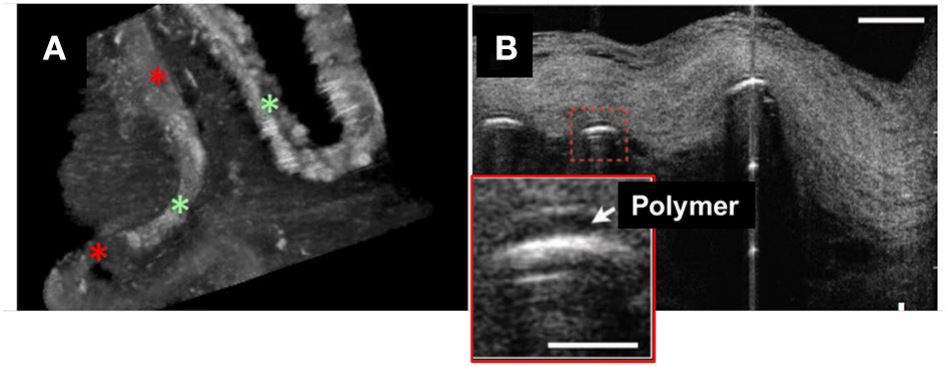
3D-μOCT of stent and endothelial coverage morphology in the human cadaver coronary artery *ex vivo*. **(A)** 3D-μOCT image of a drug-eluting stent (DES) implanted in a human coronary artery, showing tissue coverage with low intensity signal (red asterisks) and the surface of polymer coating as highly reflective regions (green asterisks). Scale bars, 100 μm. Unpublished data, obtained at the Massachusetts General Hospital. **(B)** DES struts showing polymers (red dashed box and inset) overlying the stent strut reflections. Scale bars, 30 μm. Figure and capture reprinted with permission from Liu et al. (28).

μOCT has also been used to visualize endothelial cells in human cadaver coronaries *ex vivo*. Endothelial pavementing was confirmed in early coronary lesions with intimal thickening and fibrous plaque (4) while lesions with superficial nodular calcification and necrotic core (4) lacked endothelial cells. Indeed, the results of surface roughness measurements indicated that endothelial cell distributions diminished over fibroatheromatous and fibrocalcific coronary plaques as compared with intimal-thickening and fibrous lesions (40). 3D-μOCT images of drug-eluting stents (DES) implanted in coronary segments also showed variable presence or absence of the endothelial cell coverage, which standard OCT was unable to identify (Figure 2A).

Studies have suggested that fluid shear stress induces spindle-shaped endothelial morphology that is aligned in the direction of flow while those exposed to low endothelial shear stress (ESS) are nonuniformly oriented (42). Furthermore, low and turbulent induce increased vascular permeability (36, 42) that may increase the probability of LDL and leukocyte influx. Because, it can be performed on fresh tissue and over large areas in three-dimensions, μOCT assessment of endothelial morphology's could increase our understanding of coronary regions altered by shear stress, such as bifurcations and segments at myocardial bridges (43).

Since ruptured coronary plaques account for the majority of ACS (44), an improved understanding of the role of endothelial cells in the progression of atherosclerosis and early identification of plaques at high risk are anticipated to have considerable clinical impact. A number of seminal studies have suggested that certain OCT features of thin cap fibroatheromas (TCFA), such as the thickness of fibrous caps, are critical (13, 45). However, OCT cut off values for high risk cap thicknesses are still undetermined (23). Evidence suggests that in TCFA lesions, apoptotic macrophages are not efficiently cleared by efferocytosis and are therefore prone to secondary necrosis, contributing to expansion of the necrotic core and further thinning of the fibrous cap (46). It is thus conceivable that endothelial cell wall border alignment can vary at the weakest point of these caps of TCFA. In this manner, 3D-μOCT visualization and calculation of endothelial surface roughness may augment precision definition of plaque vulnerability in humans.

Since the endothelial monolayer is below the resolution of OCT, the current OCT diagnostic criteria for a coronary erosion is defined as the presence of an intact fibrous cap at the culprit site with overlying thrombus (47). This criterion is a retrospective definition, as thrombus is required to demarcate this entity. In addition, clinically insignificant plaque erosion may occur without increased thrombogenicity resulting in healed plaque (48). Thus, there is a need for a prospective definition of a site that is at high risk of erosion and subsequent thrombus formation. Owing to its capacity to directly visualize the endothelium, μOCT may bridge these gaps in our diagnostic capabilities. Data has shown that μOCT is capable of imaging white thrombus containing fibrin (the type that is common in erosion), small platelets and multiple entrapped cells (28). Whether μOCT can clearly visualize the endothelium beneath thrombus remains an open question.

Compared to conventional OCT, which is incapable of distinctly visualizing endothelial cells, μOCT could make it possible to definitively assess endothelial coverage of stent struts and this information could be potentially used to shorten antiplatelet therapy treatment durations. In the emerging era of biodegradable-polymer DES (49) and bioresorbable scaffolds (50), μOCT should be capable of evaluating standalone stent polymers or polymer-coating overlying metal stents (Figure 2B). The use of μOCT technology to assess DES strut endothelial coverage may help resolve current questions and controversies regarding novel stent healing responses, potentially leading to a means for determining optimal antiplatelet therapy durations.

## μOCT for the Visualization of Inflammatory Cells

**Figure 3:**
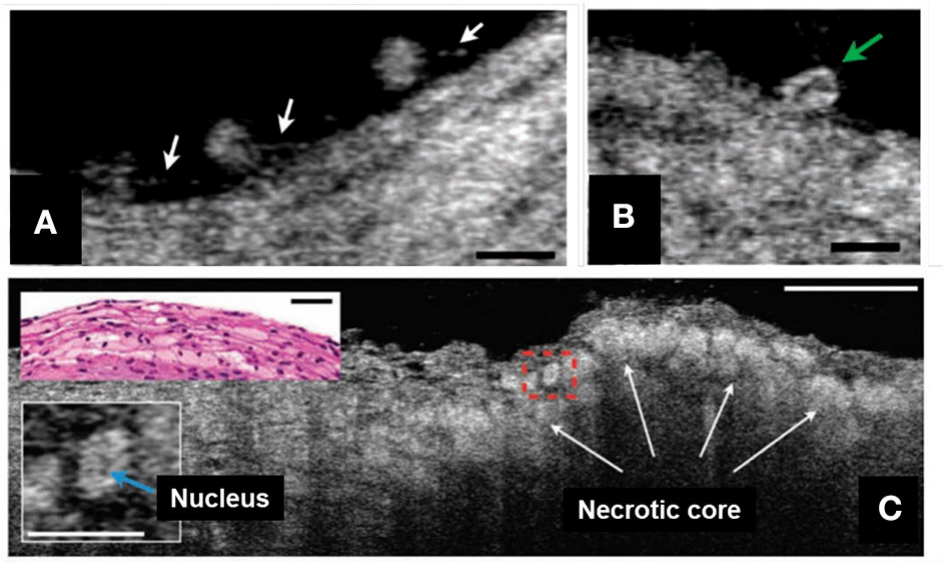
μOCT for inflammatory cell morphology in human cadaver coronaries *ex vivo*. **(A)** Multiple leukocytes tethered to the endothelial surface by linear structures, suggestive of pseudopodia (white arrows). **(B)** A cell with an indented, bean-shaped nucleus (green arrow) suggestive of a monocyte. **(C)** Necrotic core fibroatheroma with highly scattering lipid-laden macrophages or foam cells (white arrows) infiltrating the fibrous-cap that were similarly seen in the corresponding histology (top left inset). An intracellular region of low signal, a suggestive of the nucleus, was shown within the cytoplasm of a foam cell (bottom left inset, denoted by blue arrow). Scale bars, 30 μm. Figure and capture reprinted with permission from Liu et al. (28).

Inflammatory cells, such as leukocytes, monocytes, and macrophages play key roles in developing coronary atherosclerotic lesions (51). Because of its exquisite resolution, μOCT is capable of typing leukocytes based on cellular and intracellular morphology (28, 52). Additionally, μOCT has been shown to be quite capable of imaging pseudopods that inform on the activity of these cells (Figure 3A) (28). For example, compared to smaller cells with scant cytoplasm, consistent with lymphocytes, some of large cells seen on the surface had bean-shaped nucleus inside, presumably corresponding to monocytes (Figure 3B) (28). Macrophages are also seen clearly by μOCT as highly scattering, flocculent, round or ellipsoidal cells (28, 52), and are frequently observed over and within necrotic core lesions (Figure 3C) (28, 52). Some of these features have been recently demonstrated in the nasal airways *in vivo*, as μOCT was shown to be able to clearly visualize granulocytes in the mucus and epithelium of patients with cystic fibrosis (32).

Inflammatory cells play a pivotal role in all phases of coronary atherosclerosis. The compromised endothelial barrier permits them to invade into the tunica intima and initiate arterial wall thickening. Macrophages contribute to plaque vulnerability by producing proteolytic enzymes that digest extracellular matrix and destroy the integrity of the fibrous cap (46) and through their accumulation and death that form biomechanically unstable lipid deposits. For these and many other reasons, it is important to explore macrophage behavior in atherosclerotic lesions *in vivo*. Owing to its 3D imaging capabilities, μOCT makes it possible to observe such morphologic phenomena that are rarely seen in 2D cross sections. Due to the cellular resolution capabilities of μOCT, this technology could potentially also identify plaques with neutrophil extracellular trap (NETs) accumulations that induce endothelial cell apoptosis and resultant plaque erosion (53).

## μOCT for the Visualization of Intimal Crystals

**Figure 4:**
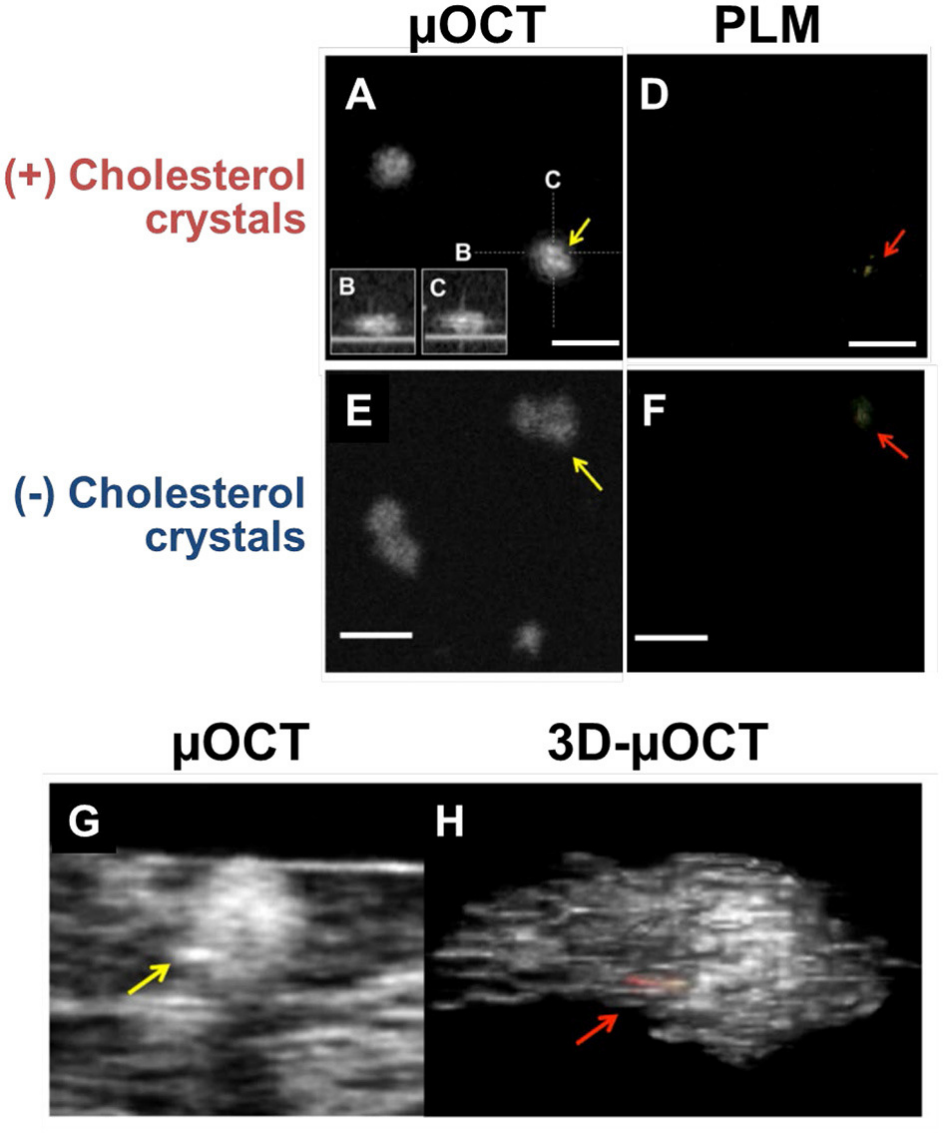
μOCT for macrophages containing cholesterol crystals *in vitro* and *ex vivo*. **(A)** *En-face* and **(B,C)** cross-sections of the macrophage demonstrated highly scattering constituents inside its cytoplasm (yellow arrow). **(D)** Corresponding polarization microscopy (PLM) confirming the cholesterol crystal inclusions (red arrow). **(E)** Cholesterol crystals that was determined by PLM **(F)** was not definitive on the μOCT image (yellow arrow). **(G,H)** Representative μOCT images of the macrophage cell in human coronary artery *ex vivo* contained highly scattering cholesterol inclusions within their cytoplasm [yellow arrow in panel **(G)**; red in panel **(H)**]. Scale bars, 50 μm. Figure and capture reprinted with permission from Kashiwagi et al. (52).

In *in vitro* cell culture experiments, macrophages containing cholesterol crystals demonstrated higher cytoplasmic scattering in μOCT images when compared to those without cholesterol crystals (Figure 4) (52). Of note, there was a discrepancy that cholesterol crystals were detected by the gold-standard polarization microscopy but were not seen on the μOCT image (Figures 4A,B). Using polarization microscopy, the accuracy of cholesterol crystal inclusions in macrophages relied on the size of cholesterol crystals (the size ≥100 mm^2^, 52% vs. <100 mm^2^, 36%, *P* < 0.05). 3D-μOCT clearly visualized a macrophage cell with the high scattering cholesterol crystal within its cytoplasm (Figure 4C).

**Figure 5:**
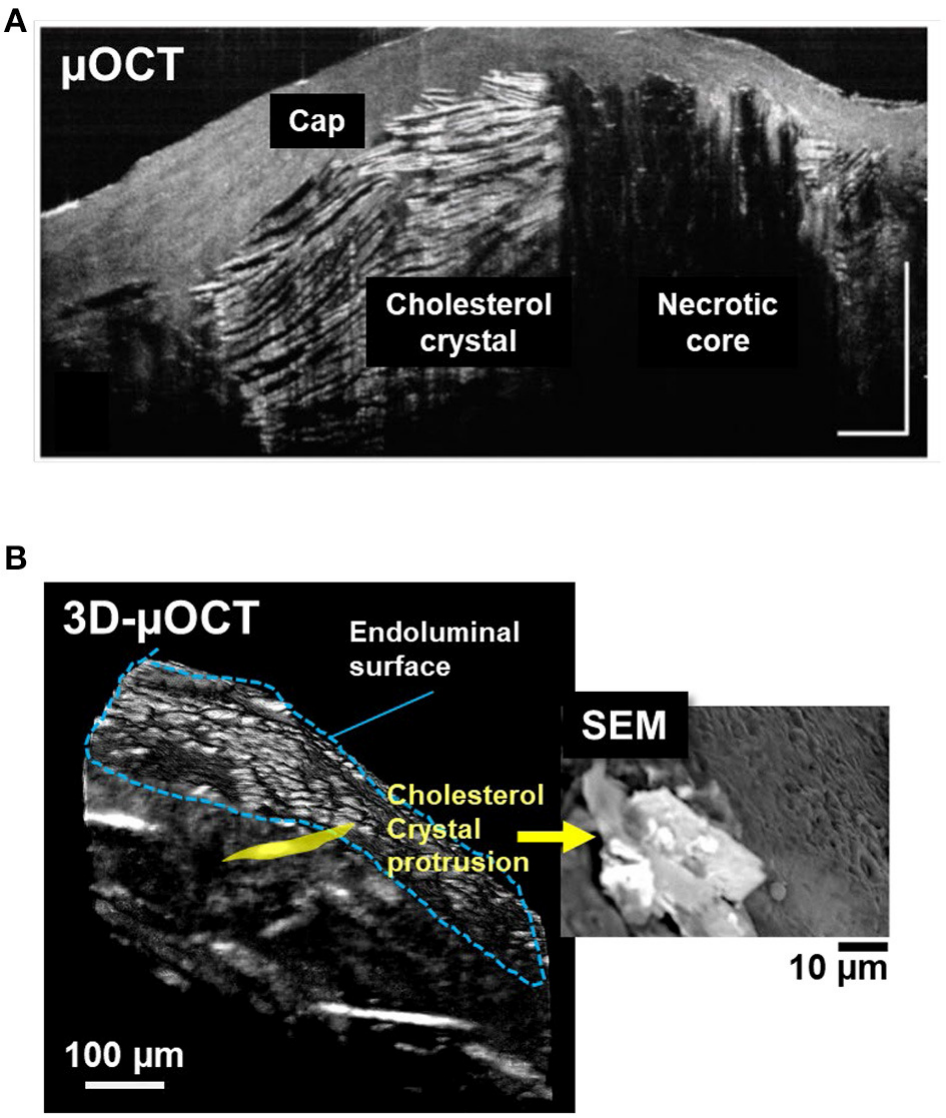
3D-μOCT for visualizing cholesterol crystals and their protrusion in the human cadaver coronary artery *ex vivo*. **(A)** Fibroatheroma with large necrotic core (NC) showing multilayered cholesterol crystals (CC), characterized by reflections from their top and bottom surfaces. Scale bars, 30 μm. Figure and capture reprinted with permission from Liu et al. (28). **(B)** 3D-μOCT for a fibroatheromatous human coronary plaque with manually segmenting a protruding cholesterol crystal (yellow area). **(B)** Corresponding SEM image showing cholesterol crystals protruding from the arterial wall. Unpublished data, obtained at the Massachusetts General Hospital.

Cholesterol crystal protrusion toward the lumen has recently been proposed as a possible cause of thrombosis and resultant ACS (54). 3D-μOCT has shown clear delineation of multilayered cholesterol crystal sheets in human cadaver coronary arteries (Figure 5A) (28) and their protrusions that were similar to what seen by SEM (Figure 5B).

Several recent studies have highlighted that anti-inflammatory pharmacotherapeutic strategies (e.g., an interleukin-1β neutralizing human monoclonal antibody, colchicine) (55, 56) may have a high potential to eliminate residual risk of CAD. Intimal crystals have been identified as a possible therapeutic target for cardiovascular disease, due to the potential of these crystals to exacerbate inflammation through inflammasome-mediated cytokine production/activation (57, 58). Identification of localized vascular inflammation as intimal crystals surrounded by inflammatory changes using μOCT would be helpful for assessing the effects of these novel therapeutic agents in patients *in vivo*.

## Catheter-Based Intravascular-μOCT

**Figure 6:**
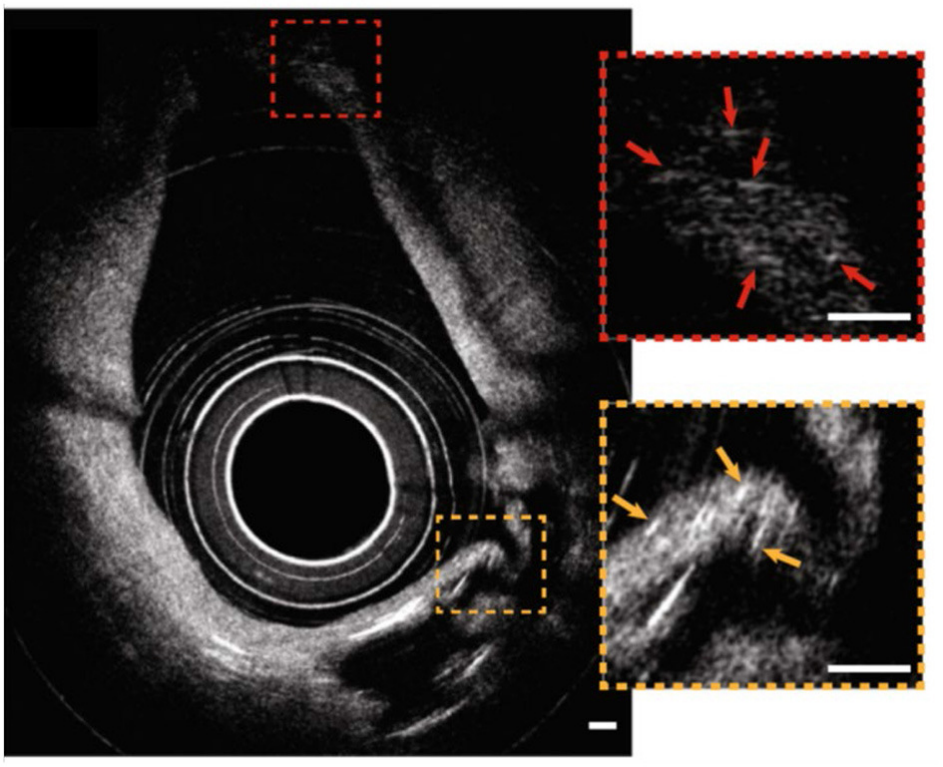
Catheter-based intravascular μOCT for cholesterol crystals in the human cadaver coronary *ex vivo*. Circumferential image showing that μOCT was capable of resolving small cholesterol crystals at distances close to the sheath (a couple hundred microns, orange inset) and far from the sheath (~1 mm, red inset) simultaneously. Scale bars, 100 μm. Figure and capture reprinted with permission from Yin et al. (35).

**Figure 7:**
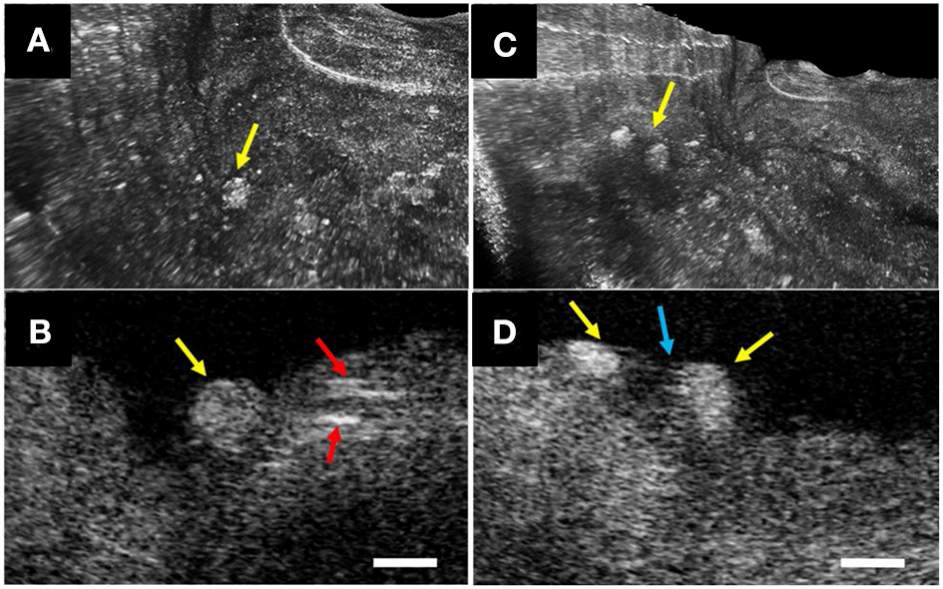
Intravascular μOCT for inflammatory cells in human cadaver coronaries *ex vivo*. **(A)** Individual macrophages (yellow arrows) residing on the surface of a fibroatheromatous plaque that **(B)** appeared to be transmigrating through the endothelium toward a deposit of intimal cholesterol crystals (red arrows). **(C)** 3D-μOCT image showing a pair of macrophages tethered to the surface, **(D)** polarized toward one another with extended pseudopodia (blue arrow). Scale bars, 50 μm. Figure and capture reprinted with permission from Yin et al. (35).

Recently, the optical imaging elements required to conduct intravascular μOCT were demonstrated (33, 34) and integrated into a catheter that had a size that was suitable for human coronary imaging (35). The imaging capability of the intravascular μOCT catheter was shown in human cadaver coronary arteries *ex vivo* and atherosclerotic rabbit aortae *in vivo* (35). μOCT circumferential views displayed cellular and subcellular coronary structures that were not readily identified by the standard OCT. For instance, small or large cholesterol crystal sheets were consistently noted in human lipid-rich plaques *ex vivo* (Figure 6) that were sometimes difficult to interpret in corresponding convention IVOCT images. As with the *ex vivo* bench top studies, smooth muscle cells could be clearly visualized as low-intensity, slit-like structures within the intima. Likewise, cross-sectional μOCT showed macrophage diapedesis in human coronaries *ex vivo*. 3D-rendering of μOCT images exhibited that individual macrophages residing on the surface of fibroatheromatous plaques that appeared to be transmigrating through the endothelium toward a deposit of intimal cholesterol crystals (Figures 7A,B) or with their pseudopods opposing each other (Figures 7C,D). Thrombus could be noted with cells that were consistent with leucocytes embedded in the intraluminal mass. The findings of this study (35) indicate that we are on the threshold of conducting intracoronary μOCT *in vivo* and await the development of clinical versions of these devices for the first-in-human studies.

## Limitations of μOCT and Technological Barriers for Clinical Applications

To achieve clinical intracoronary μOCT in the cardiac catheterization lab, several limitations and barriers still need to be resolved. First, to attain such high resolution images, μOCT is currently conducted at a shorter light wavelength (centered at 800 nm) that is lower than that of standard OCT (1,300 nm). The use of this shorter wavelength decreases the penetration depth of light in tissue, potentially further compromising its ability to assess intimal thickness and thus plaque remodeling. Second, imaging with μOCT collects ~3 orders of magnitude more data than imaging with standard OCT, and so the image sizes of a μOCT pullback will be 1,000 times greater than those of a standard OCT pullback. This 1,000-fold increase in data puts a great strain on data acquisition sensitivity and electronics, so currently the frame rate of acquiring μOCT images is significantly slower than that of standard OCT. With today's technology, imaging the entire length of a coronary artery with isotropic 1–2 μm resolution would require many pullbacks, each needing a radiocontrast flush for blood clearance. Interpreting the immense amount of information provided by μOCT may be difficult for interventional cardiologist; it is likely that artificial intelligence will be needed to aid image analysis. 3D visualization of μOCT will also likely facilitate image understanding in real time. The development of machine learning algorithms and rapid 3D rendering are ongoing topics of investigation in the μOCT field. Future technological developments will be focused on addressing these limitations to enable practical application of μOCT in the cath lab.

## Potential Scientific and Clinical Implications of μOCT

**Figure 8:**
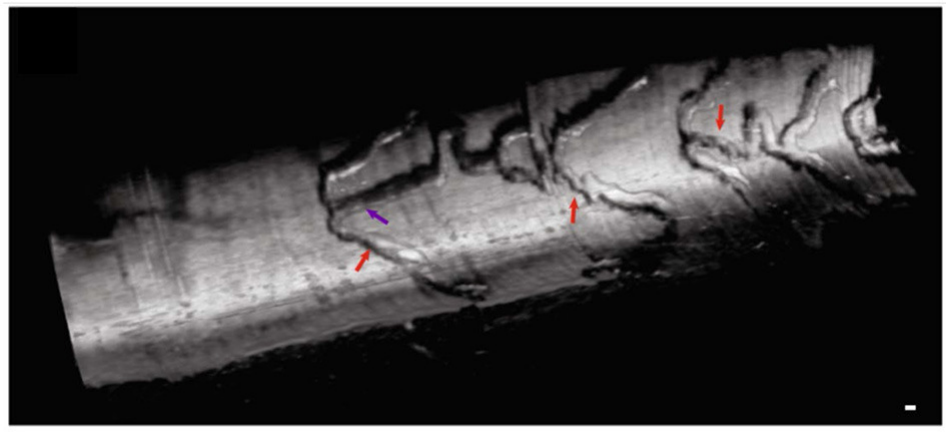
Intravascular 3D-μOCT of a drug-eluting stent implanted in the atherosclerotic rabbit iliac artery *in vivo*. The artery was imaged immediately after the stent implantation. Stent struts are denoted by the purple and red arrows. Scale bar, 100 μm. Figure and capture reprinted with permission from Yin et al. (35).

Clinical applications of μOCT for coronary imaging has the potential to be extensive. In the very beginning of coronary plaque development, altered shear-stress affects the endothelial cell alignment and effectuates endothelial dysfunction (42). Although such a relationship between shear-stress and endothelial cell orientation has been known for a long while, the finding has not been demonstrated in living patients *in vivo*. Furthermore, visualization of ongoing inflammatory cell adhesion, plaque disruption and blood coagulation remain elusive in humans *in vivo*. For interventional cardiology, μOCT will provide precise information for acute thrombotic formation around stent struts (23) at a microscopic level. A longitudinal view of stent architecture by 3D-μOCT has been demonstrated in rabbit aorta *in vivo* (Figure 8) (35). The detailed information regarding stent malapposition (59) or stent fracture (60) could help in the early detection of a precursor of procedure-related stent thrombosis. Visualization of DES polymer cracking (61) and the tissue response to anti-proliferative agents delivered by drug-coated balloons (62) could help clinicians to predict the arterial healing process.

## Conclusion

μOCT is a next-generation form of OCT that provides an order of magnitude increase in axial and lateral resolution. Our group has demonstrated that μOCT enables the visualization of structures relevant to coronary atherosclerosis pathogenesis and stent healing at cellular/subcellular levels. The recently developed μOCT coronary catheter brings this technology close to clinial use. Clinical studies will be conducted with intracoronary μOCT in the near future. Results will potentially change the landscape of coronary imaging and our understanding of coronary disease and its treatment.

## Author Contributions

All authors listed have made a substantial, direct and intellectual contribution to the work, and approved it for publication.

## Conflict of Interest

GT receives catheter materials from Terumo Corporation. Massachusetts General Hospital has a licensing arrangement with Terumo Corporation. He has the rights to receive royalties from this licensing arrangement and he receives sponsored research funding pertaining to coronary OCT from Vivolight, Canon Inc., and CN USA Biotech Holdings and AstraZeneca sponsor intracoronary μOCT research in GT's lab. GT has a financial/fiduciary interest in SpectraWave, a company developing an OCT-NIRS intracoronary imaging system and catheter. His financial/fiduciary interest was reviewed and is managed by the Massachusetts General Hospital and Partners HealthCare in accordance with their conflict of interest policies. He also has a consulting arrangement with SpectraWave. The remaining author declares that the research was conducted in the absence of any commercial or financial relationships that could be construed as a potential conflict of interest.
